# Global trends and emerging topics related to triglyceride-glucose index: A bibliometric analysis and visualization from 2000 to 2024

**DOI:** 10.1097/MD.0000000000039916

**Published:** 2024-10-04

**Authors:** Yusong Ye, Shu Huang, Ruiyu Wang, Jiao Jiang, Bei Luo, Wensen Ren, Yuan Chen, Xueqin Zhou, Xiaomin Shi, Wei Zhang, Lei Shi, Muhan Lü, Xiaowei Tang

**Affiliations:** a Department of Gastroenterology, the Affiliated Hospital of Southwest Medical University, Luzhou, China; b Nuclear Medicine and Molecular Imaging Key Laboratory of Sichuan Province, Luzhou, China; c Department of Gastroenterology, Lianshui County People’ Hospital, Huaian, China; d Department of Gastroenterology, Lianshui People’ Hospital of Kangda College Affiliated to Nanjing Medical University, Huaian, China.

**Keywords:** cardiovascular diseases, hotspots, insulin resistance, metabolic disorders, nonalcoholic fatty liver disease, triglyceride-glucose index

## Abstract

The triglyceride-glucose (TyG) index is a crucial marker of insulin resistance, as evidenced by numerous studies related to metabolic diseases. This bibliometric analysis investigates research trends associated with the TyG index over the past 24 years. We collected data on TyG index publications from January 1, 2000, to January 7, 2024, using the Web of Science database. Analysis was conducted utilizing VOSviewer, Scimago Graphica, and CiteSpace to evaluate publication metrics, citations, countries, institutions, authors, journals, and keywords. A total of 1163 publications from 354 journals authored by 6149 researchers across 60 countries were analyzed. China emerged as the leading contributor, with 654 publications (56.23%). Capital Medical University was the most productive institution, and Wu Shouling was the top author. *Cardiovascular Diabetology* was identified as the most influential journal. Key emerging research directions include the role of the TyG index as a representative marker for insulin resistance, particularly concerning insulin sensitivity; its association with body mass index and hyperuricemia; and its diagnostic and prognostic value in nonalcoholic fatty liver disease and cardiovascular conditions such as acute coronary syndrome, carotid plaque, and hypertension. Current trends favor cohort studies predominantly involving adult populations. Overall, China leads TyG index research, focusing on its connections to insulin sensitivity, body mass index, and hyperuricemia, while the index’s diagnostic and prognostic significance for nonalcoholic fatty liver disease and cardiovascular diseases represents an expanding research frontier.

## 1. Introduction

The growing burden of non-communicable diseases is a significant public health challenge with economic implications estimated to exceed $5000 per capita.^[1,2]^ Metabolic disorders, such as type 2 diabetes, hypertension, and nonalcoholic fatty liver disease (NAFLD), significantly contribute to this burden.^[3–5]^ Given the pivotal role of insulin resistance in these diseases, early detection and management are critical.^[6]^ The TyG index has emerged as a promising marker for insulin resistance and, as such, has attracted considerable attention for the early diagnosis and prognosis of metabolic disorders.^[7–9]^

To better comprehend the implications of the TyG index in metabolic health, a wealth of research has been amassed. This necessitates a systematic evaluation to quantify the impact of scholarly work, discern research patterns, and identify new investigative directions. Bibliometric and visual analyses offer valuable insights into scientific discourse, delineating relationships among keywords, authors, and themes.^[10]^ Our study aimed to conduct a bibliometric analysis of 2 decades’ worth of TyG index research to map the global trends and characterize the focal areas of study.

## 2. Materials and methods

### 2.1. Search strategy

**Figure 1.**
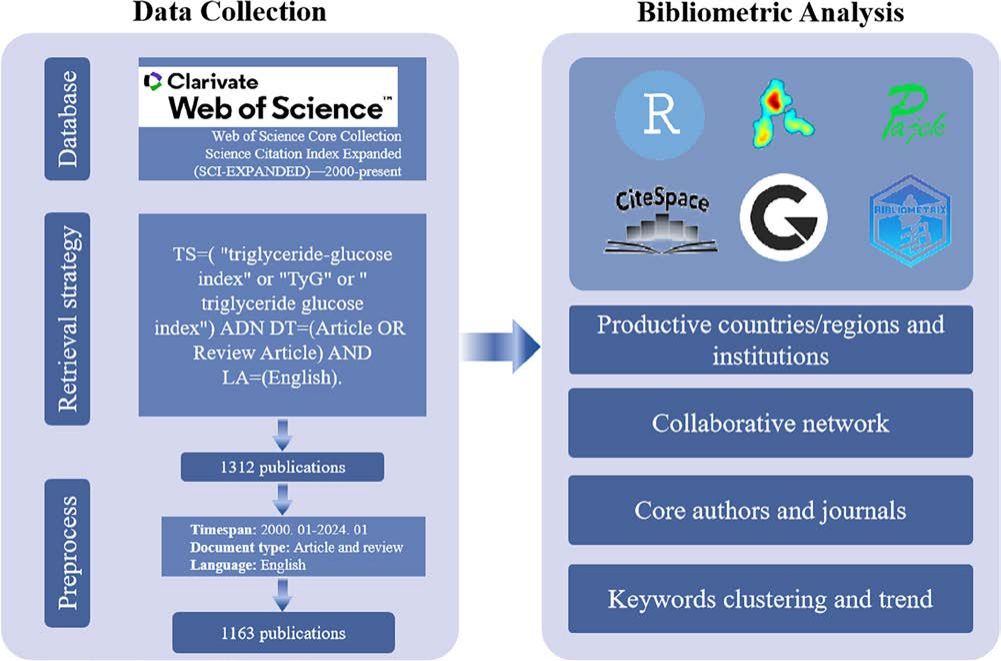
A scheme of document collection and bibliometric analysis on triglyceride-glucose index.

Comprehensive research was performed in the WoSCC on January 7, 2024. The retrieval was completed on a single day to prevent bias caused by daily database updates. The search strategy was as follows: TS=(“triglyceride-glucose index” or “TyG” or “triglyceride glucose index”) ADN DT = (Article OR Review Article) AND LA = (English). After a thorough search, we identified accurate and comprehensive articles that mentioned TyG index. The timespan of publications from January 1, 2000, to January 7, 2024. Additionally, only articles and review articles were included, and the language was limited to English (Fig. 1).

### 2.2. Data extraction

All of the information about the related publications identified by the search strategy from the WoSCC on January 7, 2024, was downloaded in txt, tab, BibTex and Excel formats for further analysis; the main search terms included title, author, country, institution, journal, keywords, and times cited count. The 2023 Journal Citation Report category quartiles of the most productive journals on TyG index were collected from the Web of Science.

### 2.3. Bibliometric analysis

The bibliometric data were analyzed using VOSviewer (version 1.6.18), the Scimago Graphica Setup (version 1.0.34), and CiteSpace (6.1. R6). VOSviewer was used to identify the number of publications and citations of the most productive countries, authors, institutions, journals, and keywords. CiteSpace was used for generating the timeline graph of the keywords. The co-authorship of countries, institutions, authors, and keywords was generated by VOSviewer and the Scimago Graphica Setup.

## 3. Results

### 3.1. Specific productivity by year and subject area

A systematic search of the WoSCC was conducted for literature on the TyG index from January 1, 2000, to January 7, 2024. From the initial 1312 articles identified, the inclusion criteria were refined to consider only original and review articles published in English, resulting in 1163 relevant publications (1130 original articles and 33 reviews).

**Figure 2.**
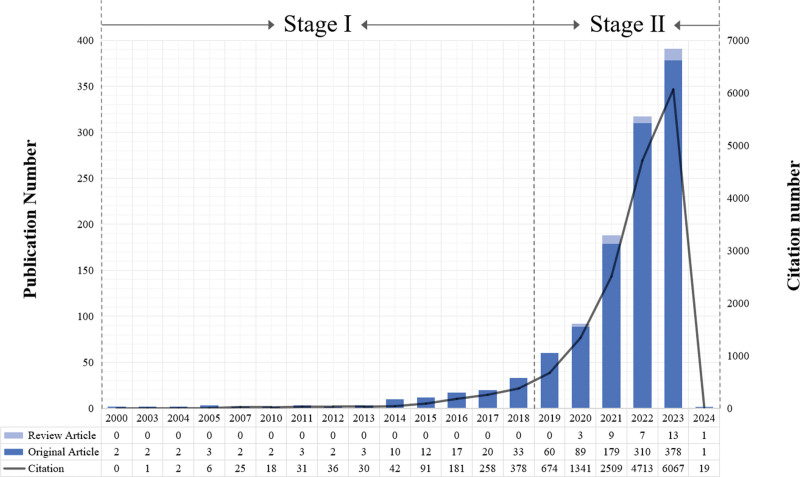
(A) Annual number of publications on triglyceride-glucose index. (B) Annual number of citations on triglyceride-glucose index.

The temporal distribution of publications was divided into 2 phases (Fig. 2): phase I (2000–2018) characterized by foundational research with an average of 8.07 articles per year and 78.50 citations per article, and phase II (2019–2024) marking a rapid growth in interest, averaging 175 articles and 2553.83 citations annually. The publication count in phase II was 21.69 times greater than in phase I, while citation rates were 32.53 times higher in phase I. Notably, data collection in early 2024 omits articles published and cited within the year, presumably under representing phase II publication and citation numbers.

### 3.2. Leading countries

**Figure 3.**
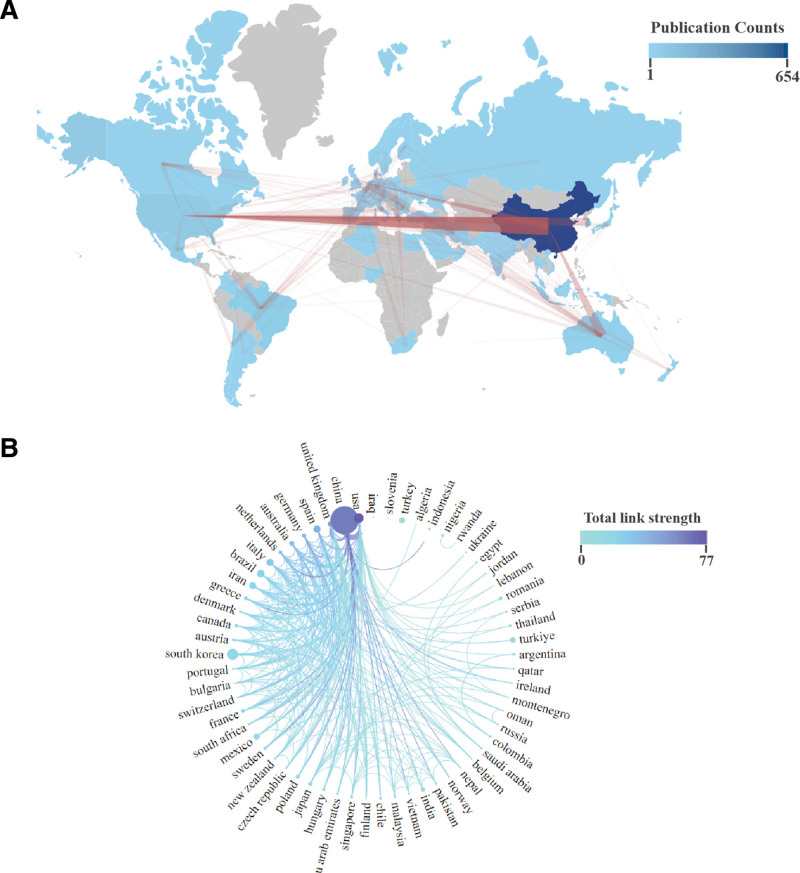
(A) Geographical distribution map of publications between countries on triglyceride-glucose index. (B) The countries collaboration network on triglyceride-glucose index.

Figure 3A provides a comprehensive overview of the global contributions of TyG index to the field, encompassing 60 countries from 6 continents. It can be observed that China holds an absolute advantage in this field, with the remaining countries having relatively small differences in publication volume. Table 1 shows the countries that have made notable contributions, with China leading as the most productive country (n = 654, 56.23%), accounting for over half of the publications in this field, followed by the South Korea (n = 99, 8.51%), USA (n = 74, 6.36%), Turkey (n = 55, 4.73%), and Brazil (n = 46, 3.96%). Notably, while China had achieved the highest total number of citations (6996), Mexico, the 10th country in terms of publication volume, exhibited the highest average citations per study (39.97).

**Table 1:** The most productive countries in terms of number of publications on triglyceride-glucose index.

Country	Counts, n (%)	Total citations	Average citations	Total link strength
China	654 (56.23%)	6996	10.70	68
South Korea	99 (8.51%)	2512	25.37	16
USA	74 (6.36%)	1852	25.03	77
Turkey	55 (4.73%)	134	2.44	2
Brazil	46 (3.96%)	802	17.43	24
Italy	43 (3.70%)	885	20.58	25
Spain	42 (3.61%)	947	22.55	36
Iran	40 (3.44%)	365	9.13	17
Mexico	31 (2.67%)	1239	39.97	10

In assessing international cooperation, an analysis was performed on all the countries contributing to development in this field (Fig. 3B). International collaboration analysis revealed the USA as the leader, followed by China and the UK, underscoring the influence of these nations in TyG research through their scholarly output, citations, and cooperative efforts.

### 3.3. Active institutions

**Figure 4.**
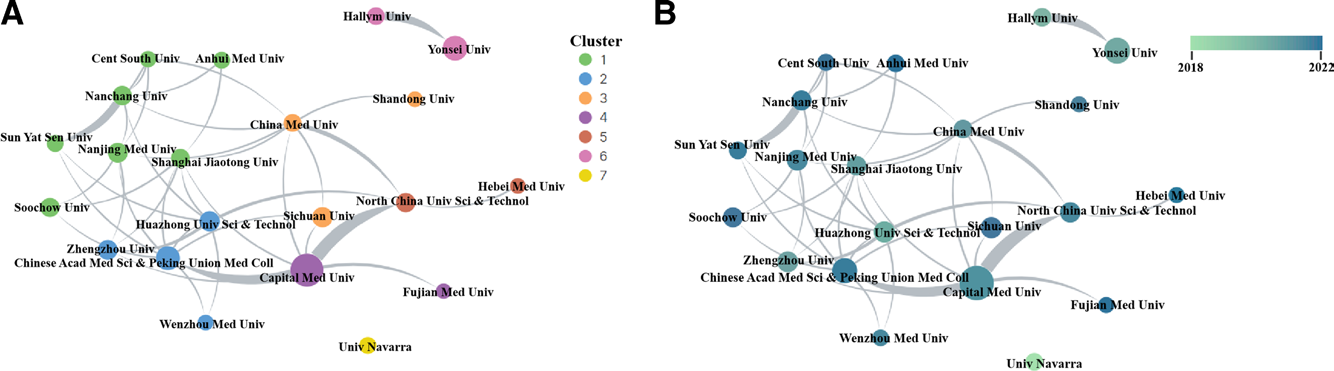
(A) The network visualization of institutions on triglyceride-glucose index. (B) The overlay visualization of institutions on triglyceride-glucose index.

Research on the TyG index involved 1483 institutions, with the top 10 accounting for 27.09% of total publications (Table 2). The Capital Meicical University in China was the most prolific (68, 5.85%), followed by Yonsei university in South Korea (39, 3.35%), and Chinese Academy of Medical Science and Peking Union Medical College (36, 3.10%). Figure 4A illustrates the collaboration among institutions with a publication count of 15 or more. The collaboration patterns reveal a robust network primarily among Chinese institutions, with the Capital Medical University leading in collaborations. Not only does the Capital Medical University stand out in terms of publication quantity, but it also holds a prominent position in collaborative efforts, making it an influential entity in this field. Figure 4B presents an overlay visualization of the TyG index, demonstrating the increasing interest of Chinese institutions in TyG in recent years.

**Table 2:** The most productive institutions and authors in terms of number of publications on triglyceride-glucose index.

Institution	Counts, n (%)	Total citations	Author	Counts, n (%)	Total citations
Capital Medical University	68 (5.85%)	938	Wu Shouling	21 (1.81%)	364
Yonsei University	39 (3.35%)	1037	Chen Shuohua	14 (1.20%)	298
Chinese Academy of Medical Sciences and Peking Union Medical College	36 (3.10%)	350	Tian Xue	12 (1.03%)	245
Huazhong University of Science and Technology	26 (2.24%)	857	Wang Anxin	11 (0.95%)	252
Sichuan University	26 (2.24%)	109	Guerrero-Romero Fernando	11 (0.95%)	961
Zhengzhou University	25 (2.15%)	481	Simental-Mendia Luis e.	11 (0.95%)	998
Najing University	25 (2.15%)	153	Zuo Yingting	10 (0.86%)	264
North China University of Science and Technology	24 (2.06%)	404	Wang Yongjun	10 (0.86%)	218
Nanchang University	23 (1.98%)	262	Chen Szu-chia	10 (0.86%)	194
Soochow University	23 (1.98%)	210	Lee Hye sun	9 (0.77%)	225

### 3.4. Active authors

**Figure 5.**
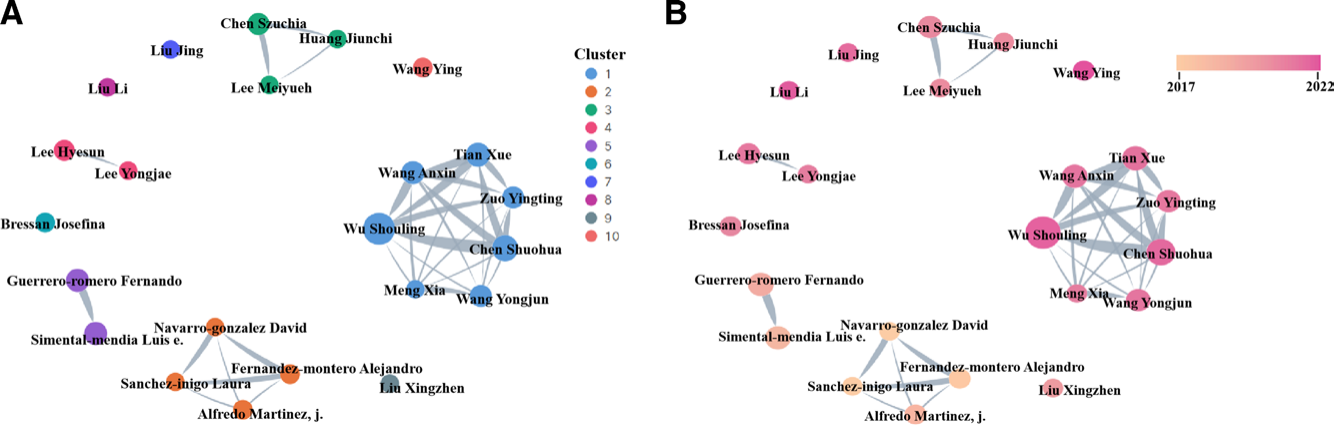
(A) The network visualization of authors on triglyceride-glucose index. (B) The overlay visualization of authors on triglyceride-glucose index.

A total of 6419 authors contributed to the TyG index literature over 24 years, with the top 10 authors – predominantly from China and Mexico – responsible for 10.23% of the publications (Table 2). Wu Shouling was the most productive author (21, 1.81%), followed by Chen Shuohua (14, 1.20%), and Tian Xue (12, 1.03%). All 3 of these top authors are from China. The Scimago Graphica Setup was employed to conduct cluster analysis of co-authorship among these authors. In Figure 5A, we can observe that cluster 1 exhibits the highest level of collaboration, with the 3 most prolific authors also belonging to this cluster. This suggests that the leading cluster of authors shows significant collaborative ties and influence. Furthermore, cluster 1 has shown significant interest in the field in recent years. This, to some extent, indicates that the authors of cluster 1 have a certain influence in TyG research (Fig. 5B).

### 3.5. Core journals

**Figure 6.**
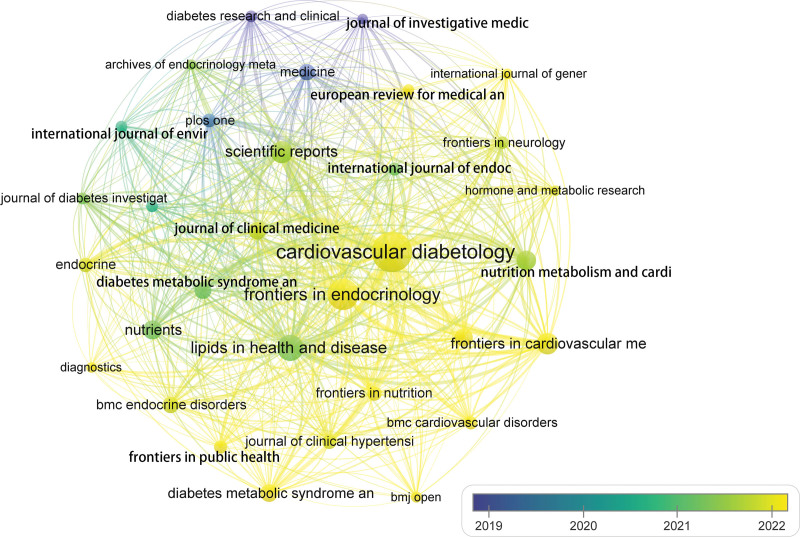
The overlay visualization of journals on triglyceride-glucose index.

Overall, 354 journals have published papers on the TyG index over the past 24 years, with the top 10 contributing 38.69% of the total (Table 3). The most prolific journal was *Cardiovascular Diabetology* (133, 11.44%), followed by *Frontiers in Endocrinology* (74, 6.36%), and *Lipids in Health and Disease* (50, 4.30%). *Cardiovascular Diabetology* was the most influential journal, with a remarkable record of productive publications and the highest impact factor of 9.3 in 2023. Figure 6 presents the overlay visualization of journals. In recent years, prominent journals such as *Cardiovascular Diabetology*, *Frontiers in Endocrinology*, and *Journal of Clinical Medicine* have demonstrated a keen interest in the TyG index.

**Table 3:** The top 10 most productive journals in terms of number of publications on triglyceride-glucose index.

Journal	Papers	Country	Total citations	Average citations	*Q*	IF
*Cardiovascular Diabetology*	133 (11.44%)	UK	3289	24.73	1	9.3
*Frontiers in Endocrinology*	74 (6.36%)	Switzerland	264	3.57	1	5.9
*Lipids in Health and Disease*	50 (4.30%)	UK	877	17.54	2	4.5
*Diabetes Metabolic Syndrome and Obesity-Targets and Therapy*	38 (3.27%)	Netherlands	217	5.71	3	3.3
*Scientific Reports*	33 (2.84%)	UK	414	12.55	2	4.6
*Frontiers in Cardiovascular Medicine*	31 (2.67%)	Switzerland	306	9.87	2	3.6
*Nutrition Metabolism and Cardiovascular Disease*	26 (2.24%)	Italy	372	14.31	2	3.9
*Nutrients*	24 (2.06%)	Switzerland	273	11.38	1	5.9
*Diabetology & Metabolic Syndrome*	21 (1.81%)	Brazil	190	9.05	2	4.8
*Journal of Clinical Medicine*	20 (1.72%)	Switzerland	177	8.85	2	3.9

### 3.6. Core keywords

**Figure 7.**
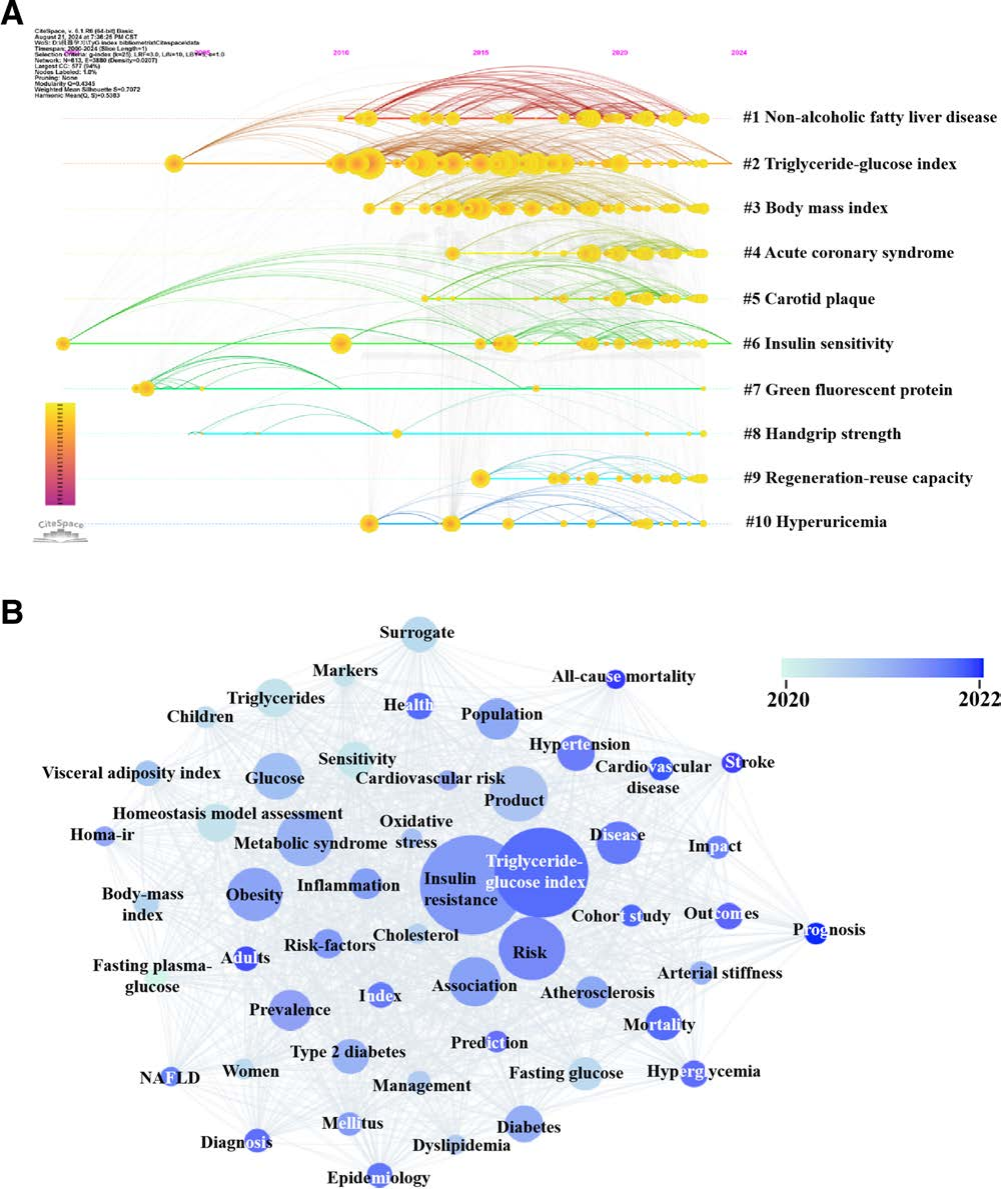
(A)The timeline view of keywords on triglyceride-glucose index. (B) The overlay visualization of keywords on triglyceride-glucose index.

Analysis of 1163 papers revealed 3256 unique keywords, with the most prevalent being “insulin resistance,” “risk,” “metabolic syndrome,” “obesity,” and “product” (Table 4). To identify emerging topics related to the TyG index, we employed 2 different bibliometric software tools for analysis (Fig. 7). Using CiteSpace, we identified the top 10 most active research themes in the TyG field (Fig. 7A), which include: #0 nonalcoholic fatty liver disease, #1 triglyceride-glucose index, #2 body mass index (BMI), #3 acute coronary syndrome, #4 carotid plaque, #5 insulin sensitivity, #6 green fluorescent protein, #7 handgrip strength, #8 regeneration-rescue capacity, and #9 hyperuricemia. The VOSviewer software identified keywords in the TyG field with research frequency exceeding 30 occurrences (Fig. 7B), with current research hotspots including “triglyceride-glucose index,” “cardiovascular disease,” “NAFLD,” “hyperuricemia,” “hypertension,” “stroke,” “adults,” “cohort study,” “prognosis,” “diagnosis,” “mortality,” “outcomes,” and “all-cause mortality.” Based on the analysis of TyG research hotspots from the 2 bibliometric tools, we summarized the emerging research directions as follows: the role of TyG index as a representative marker for insulin resistance, especially in relation to insulin sensitivity. the association between TyG index and BMI. the association between TyG index and hyperuricemia, the diagnostic and prognostic value of TyG index in liver disease (specifically NAFLD), cardiovascular diseases (specifically acute coronary syndrome, carotid plaque, and hypertension). Additionally, from the perspective of research methods and target population, the current trend in TyG-related research leans towards utilizing cohort studies predominantly involving adult populations.

**Table 4:** The top 10 keywords with the highest number of occurrences on triglyceride-glucose index.

Keywords	Occurrences
Insulin resistance	805
Triglyceride-glucose index	660
Risk	328
Product	255
Metabolic syndrome	242
Obesity	227
Association	198
Glucose	178
Disease	148
Population	139

## 4. Discussion

This study employed bibliometric and visual analytic techniques to scrutinize the evolution of research on the TyG index over the last quarter-century. Our analysis indicates a rapid growth in publications, predominantly driven by contributions from Asian nations. Research on TyG is currently concentrated on 4 main themes: its role as an insulin resistance marker, its association with BMI, its correlation with hyperuricemia, and its diagnostic and prognostic relevance in liver and cardiovascular diseases.

The TyG index, derived from fasting triglycerides and glucose, is a potent surrogate for insulin resistance and a predictor for metabolic disorders such as type 2 diabetes, obesity, hypertension, and cardiovascular diseases.^[11–17]^ While the hyperinsulinemic-euglycemic clamp (HIEC) remains the gold standard for measuring insulin resistance, its invasiveness, complexity, and cost limit its practicality for broad application.^[7,18]^ Consequently, the TyG index emerges as a viable, noninvasive alternative with established efficacy for insulin resistance assessment in clinical and epidemiological settings.^[19–21]^

What are the current research trends on TyG index? Bibliometric analysis reveals a substantial rise in TyG index research, with publication volume increasing 195.5-fold since 2000 and citations soaring to 6067 times the 2000 figure. Notably, 90% of the most-cited papers appear in high-impact Q1 or Q2 journals, underscoring TyG’s escalating relevance in metabolic disease studies. China, South Korea, and the USA lead in publication output, with China showing marked progress, yet still trailing the USA in highly cited works, indicating room for growth in impactful research.

Strong networks among authors, institutions, and nations are vital for academic progress. China has become a prominent publisher in TyG index research, engaging significantly in international collaborations, especially with the USA. Chinese researchers, however, favor domestic over international partnerships, possibly due to language barriers and regional variations in metabolic disease patterns. Nevertheless, broader international cooperation could greatly benefit academia by enhancing communication, data sharing, and resource integration, thereby increasing the research’s impact and addressing global health issues more effectively. Promoting such collaborations is essential for advancing TyG index research and achieving wider academic and societal advancements.

Keywords are pivotal, distilling the essence and conclusions of research papers. Analyzing keywords across studies provides insight into the field’s status and trends. In this study, a comprehensive analysis of keywords was conducted, resulting in the identification of a total of 3256 distinct keywords, of which 167 appeared more than 10 times. Results indicate that current TyG index research focuses on its role in indicating insulin resistance and its diagnostic and prognostic relevance to metabolic disorders like NAFLD and cardiovascular diseases. Moreover, cohort studies are becoming the prevalent research method in this area.

Cardiovascular disease is the leading cause of death worldwide among chronic diseases. The development of atherosclerotic plaques in arteries plays a crucial role in the pathogenesis of serious cardiovascular conditions such as coronary heart disease and myocardial infarction.^[22]^ Recent studies have shown that insulin resistance is implicated in the formation and remodeling of coronary artery atherosclerotic plaques, independent of traditional risk factors such as age, smoking, and hypertension.^[23]^ Thus, insulin resistance assessment is vital for cardiovascular disease management. The TyG index has gained recognition in cardiovascular research as a reliable marker for disease diagnosis and progression. Li et al associated high and variable TyG index levels with increased cardiovascular risk.^[24]^ Wang et al suggested that TyG, as an insulin resistance proxy, could predict coronary artery disease severity, especially in prediabetics.^[25]^ Liu et al demonstrated a significant association between higher baseline levels of TyG and an increased risk of future cardiovascular disease in postmenopausal women.^[26]^

NAFLD, a metabolic liver syndrome characterized by insulin resistance, is now the most prevalent liver disease due to lifestyle shifts and rising obesity rates, leading to chronic liver conditions.^[27–29]^ With a global NAFLD prevalence of 25%, especially in Asia and the Middle East, and most patients asymptomatic with no approved treatments, early detection is imperative.^[30]^ The TyG index, a cost-effective proxy for insulin resistance, has been linked to NAFLD development. Xue et al confirmed its association with NAFLD and liver fibrosis risk.^[31]^ Ye et al identified the TyG index as a diagnostic biomarker for NAFLD, enhanced when combined with ALT.^[32]^ Wang et al’s meta-analysis affirmed the TyG index’s diagnostic and predictive utility for NAFLD.^[33]^

However, despite the TyG index’s relevance in metabolic disease prediction and diagnosis, it faces challenges. It does not distinguish between specific insulin resistance types, such as muscle insulin resistance and adipose tissue insulin resistance, limiting the understanding of underlying mechanisms. Additionally, a standardized threshold value for the TyG index is yet to be established, with variations across studies. Moreover, the TyG index is not a standalone diagnostic tool; it requires integration with other clinical metrics for precise diagnosis and prognosis in practice. Additionally, Most TyG index research relies on cross-sectional or medium-term longitudinal data, which is limited in establishing a definitive long-term predictive relationship with metabolic diseases. Our study indicates that cohort studies are now pivotal for investigating the TyG index-disease relationship, with their extended observations expected to enhance the TyG index’s predictive accuracy.

This study surveys global TyG index research trends, noting a surge in publications and a focus on its association with insulin resistance, BMI, hyperuricemia, and its role in metabolic disease diagnosis and prognosis. Future research will translate these insights into clinical practice, potentially refining treatment and patient outcomes. This work offers a valuable framework for future TyG index investigations.

We acknowledge that this study has several limitations. First, the data were extracted entirely from WoSCC and did not include other databases, such as PubMed and Embase, which may have resulted in incomplete results. However, the WoSCC is generally considered the most commonly used database in bibliometric analysis because it can update citation networks in a timely and comprehensive manner. Second, due to the different algorithms applied by different analysis software programs, the amount of relevant information calculated in some articles is slightly different, and some emerging topics related to TyG index may not be determined. The further development of bibliometric analysis methods can help to address these limitations.

## 5. Conclusion

China leads TyG index research, with a focus on its association with insulin sensitivity, BMI, and hyperuricemia. The index’s diagnostic and prognostic importance for NAFLD and cardiovascular diseases has become a research frontier. Cohort studies in adults dominate current research trends.

## Author contributions

**Conceptualization:** Yusong Ye, Ruiyu Wang, Yuan Chen.

**Data curation:** Yusong Ye, Wensen Ren, Bei Luo, Lei Shi.

**Formal analysis:** Yusong Ye, Xiaomin Shi.

**Methodology:** Yusong Ye, Shu Huang, Wensen Ren, Jiao Jiang.

**Resources:** Yusong Ye, Shu Huang, Wei Zhang.

**Software:** Yusong Ye, Shu Huang, Wensen Ren, Ruiyu Wang.

**Supervision:** Muhan Lü, Xiaowei Tang.

**Validation:** Shu Huang, Xueqin Zhou, Wei Zhang.

**Visualization:** Yusong Ye, Wensen Ren, Xueqin Zhou.

**Writing – original draft:** Yusong Ye.

**Writing – review & editing:** Muhan Lü, Xiaowei Tang.
